# Four-Rod Stabilization of Severely Destabilized Lumbar Spine Caused by Metastatic Tumor

**DOI:** 10.1155/2013/254684

**Published:** 2013-06-01

**Authors:** Isao Shibuya, Koichi Sairyo, Yasuo Kanamori, Akira Dezawa

**Affiliations:** Department of Orthopedic Surgery, Teikyo University Mizonokuchi Hospital, 3-8-3 Mizonokuchi, Takatsu-ku, Kawasaki 213-8507, Japan

## Abstract

We report a case of a 67-year-old female with severely destabilized lumbar spine caused by metastatic malignant tumor. The primary lesion was a thyroid follicular adenocarcinoma. Complete destruction of the L3, L4, and L5 vertebrae had resulted in severe instability, which left the patient with severe back pain and bed-ridden. Since the vertebrae were so severely damaged at 3 levels, 4 rods were used to stabilize the spine. Following stabilization, the pain was alleviated and the patient's quality of life improved. We introduce here the 4-rod technique to stabilize the spine over 3 vertebral levels following severe destruction by metastatic tumor.

## 1. Introduction

Metastatic spinal tumor is not uncommon in these days. It is well accepted and has been demonstrated that the primary tumor site is the most important prognostic factor for survival [1–3]. If the primary tumor is not aggressive, frequently surgical reconstruction is selected to improve quality of life of the patients. We experienced severely destructed spinal metastasis, which L3-L4-L5 showed totally osteolytic destruction. For such case, we felt that stabilization using two rods is not enough rigid, and we used 4 rods instead. In this case report, we introduce the case and our stabilization technique using 4 rods. 

## 2. Case Presrntation

**Figure 1:**
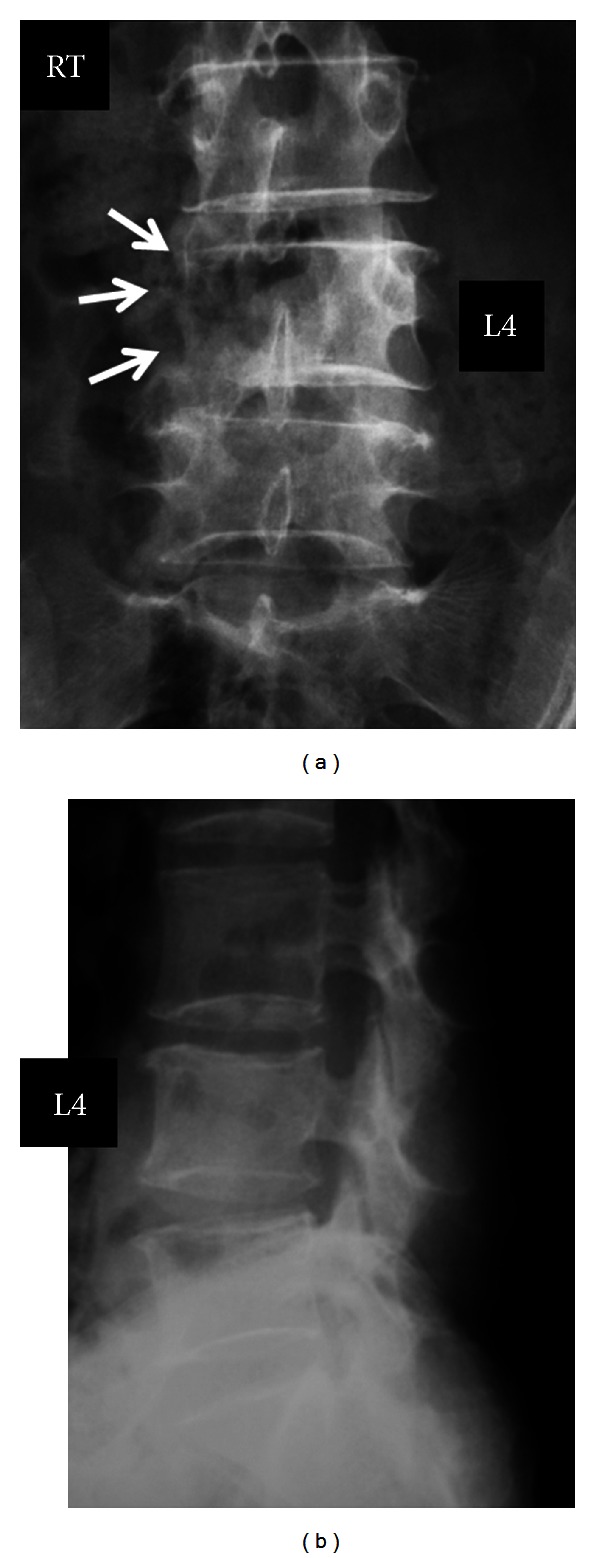
Plain radiographs (June 2006) at first consultation clearly show bone destruction of the right L4 pedicle as the winking owl sign, which suggests a metastatic malignant tumor of the spine.

**Figure 2:**
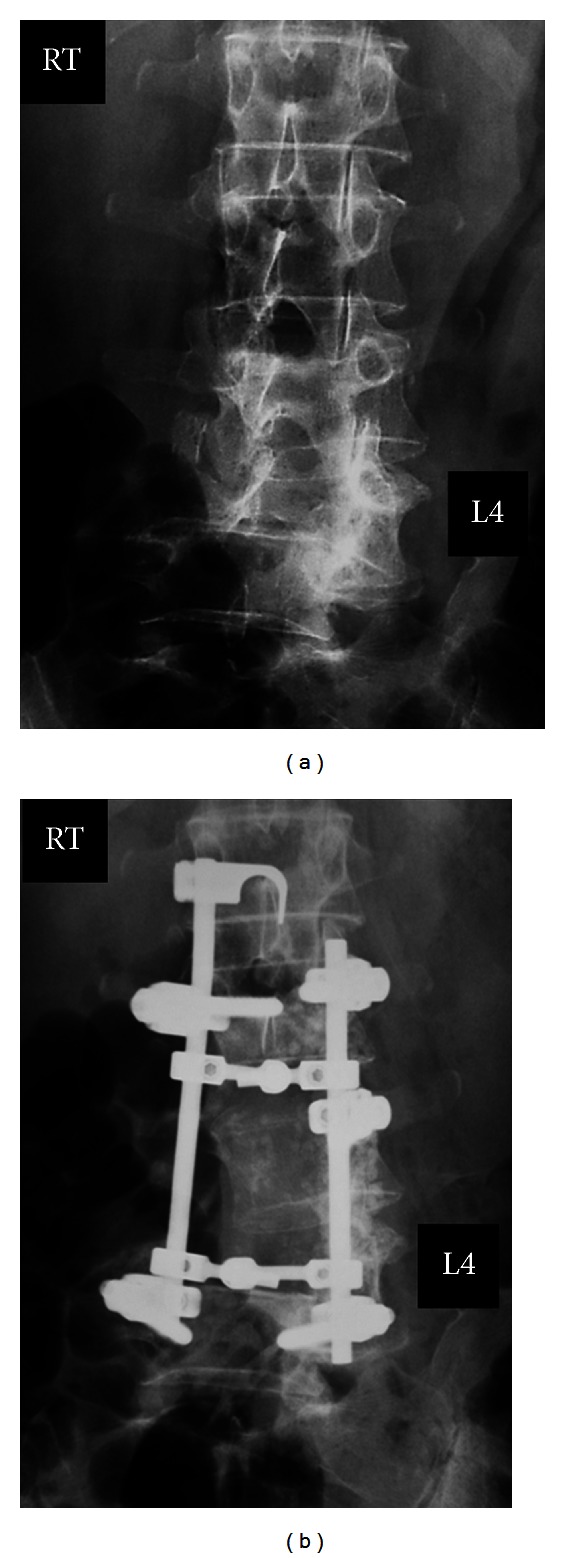
Plain radiographs before and after the first surgery (January 2007). Six months after the first consultation, the tumor size was increased and had invaded the entire L4 and right L3 vertebral body (a). Spine reconstructive surgery was conducted using a pedicle screw, hook-and-rod system (b).

A 67-year-old female presented to our clinic with a complaint of right leg pain in June 2005. An anteroposterior plain radiograph (Figure 1) clearly showed bone destruction in the right L4 pedicle as the winking owl sign, suggesting metastatic malignant tumor of the spine. Needle biopsy conducted for histological examination revealed moderately differentiated adenocarcinoma. Following an extensive general examination, a cervical mass was identified in the thyroid gland, and aspiration biopsy of the thyroid tumor exhibited similar histologic findings to the tissue from the metastatic spinal lesion. Accordingly, the patient was diagnosed with primary thyroid adenocarcinoma metastatic to the lumbar spine. Following subsequent resection of the primary tumor, radiculopathy was not so severe and the pain was well controlled with medication. She was able to walk independently and complained only of tingling in the right lower leg. Following primary tumor resection, the metastatic mass and osteolytic area gradually increased in size. Radiotherapy was selected to control the metastatic tumor, and the patient received I-131 NoI 3.7 GBq orally. In January 2007, she complained of severe back pain and weakness of the right leg. The tumor was found to have invaded the entire L4 and right L3 vertebral body (Figure 2(a)). Table 1 summarizes the neurological findings. 

**Figure 3:**
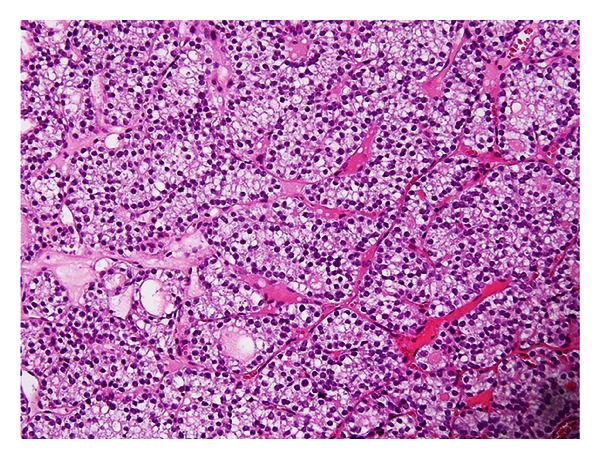
Histological findings of the spinal metastatic tumor were compatible with the primary thyroid follicular adenocarcinoma.

Lumbar spine reconstructive surgery was conducted using a pedicle screw, hook-and-rod system (Figure 2(b)). Following laminectomy of L2 to L5, as much as possible of the metastatic tumor compressing the neural tissue was removed, and pedicle screws were inserted at bilateral L2, left L3, and bilateral L5. A supralaminar hook was also used. Motor function was improved postoperatively and she was able to walk without support. Histologic findings of the spinal metastatic tumor were compatible with the primary thyroid follicular adenocarcinoma (Figure 3). After the surgery, supportive treatment including chemotherapy and radiotherapy was not effective in controlling the growth of the metastatic tumor.

In September 2009, the patient complained of weakness in the left leg and started using a cane when walking. In November, she was no longer able to walk and started using a wheelchair. There was gradual aggravation of the neurological findings, and in February 2010 the patient was immobile due to severe pain, which could not be alleviated even by narcotics. The neurological results at that time are given in Table 2. 

**Figure 4:**
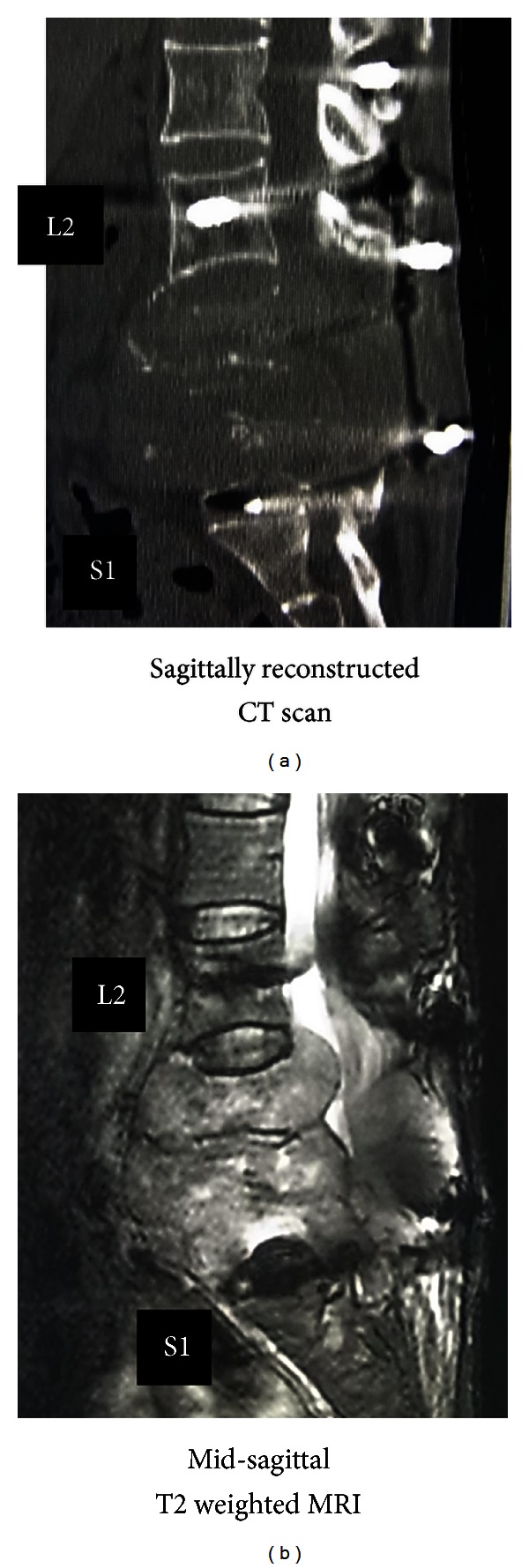
CT and MRI before the second surgery (February 2010), around 3 years after the first surgery. Sagittally reconstructed CT images (a) revealed complete destruction by the tumor of the L3, L4, and L5 vertebrae including the vertebral bodies and laminae.

**Figure 5:**
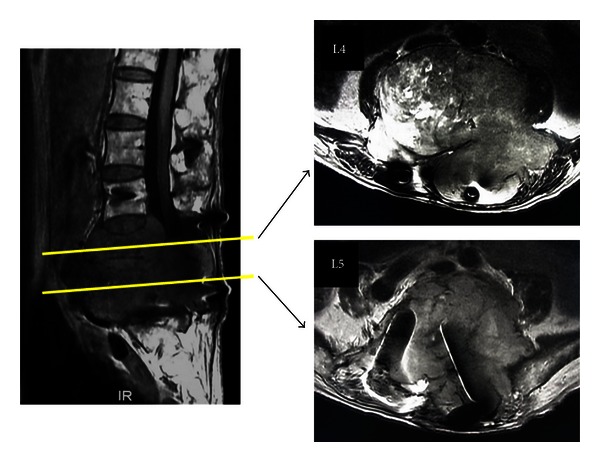
MRI before the second surgery (February 2010) revealed a large mass compressing the neural tissue at the L3, L4, and L5 levels.

**Figure 6:**
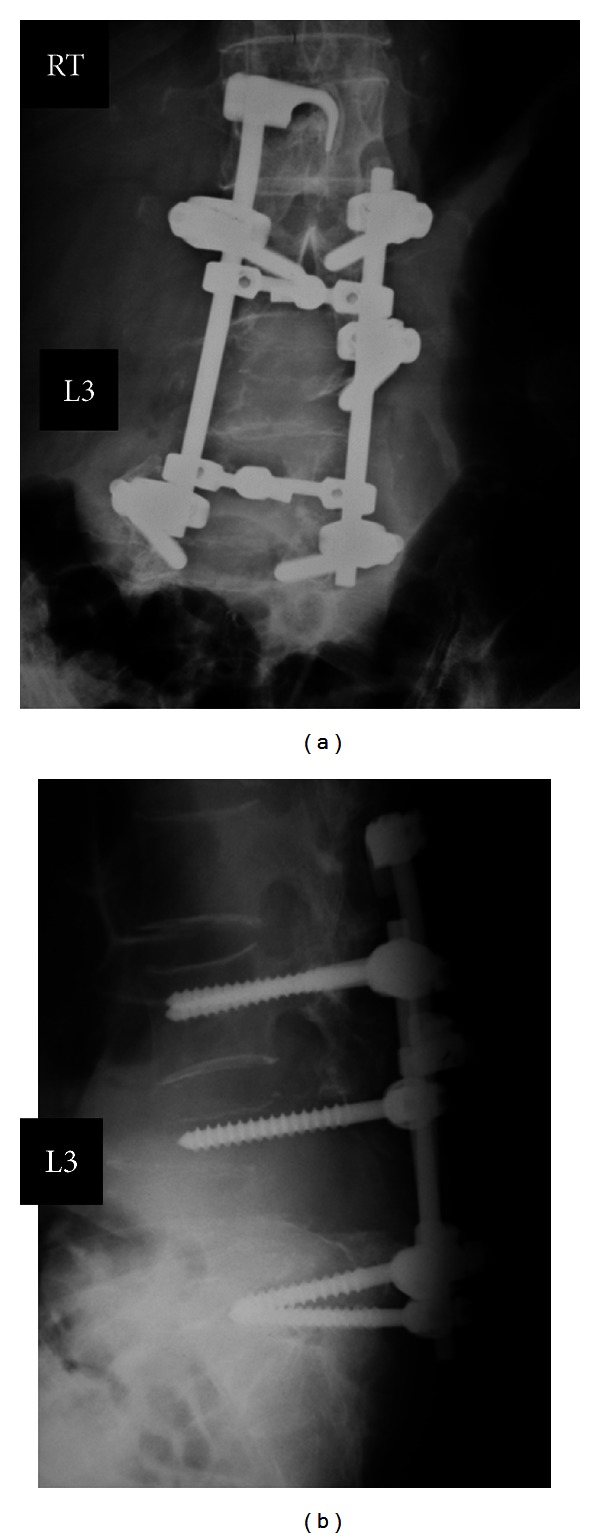
Plain radiographs before the second surgery (February 2010) show that the pedicle screws inserted at L3 and L5 had completely loosened and were displaced.

**Figure 7:**
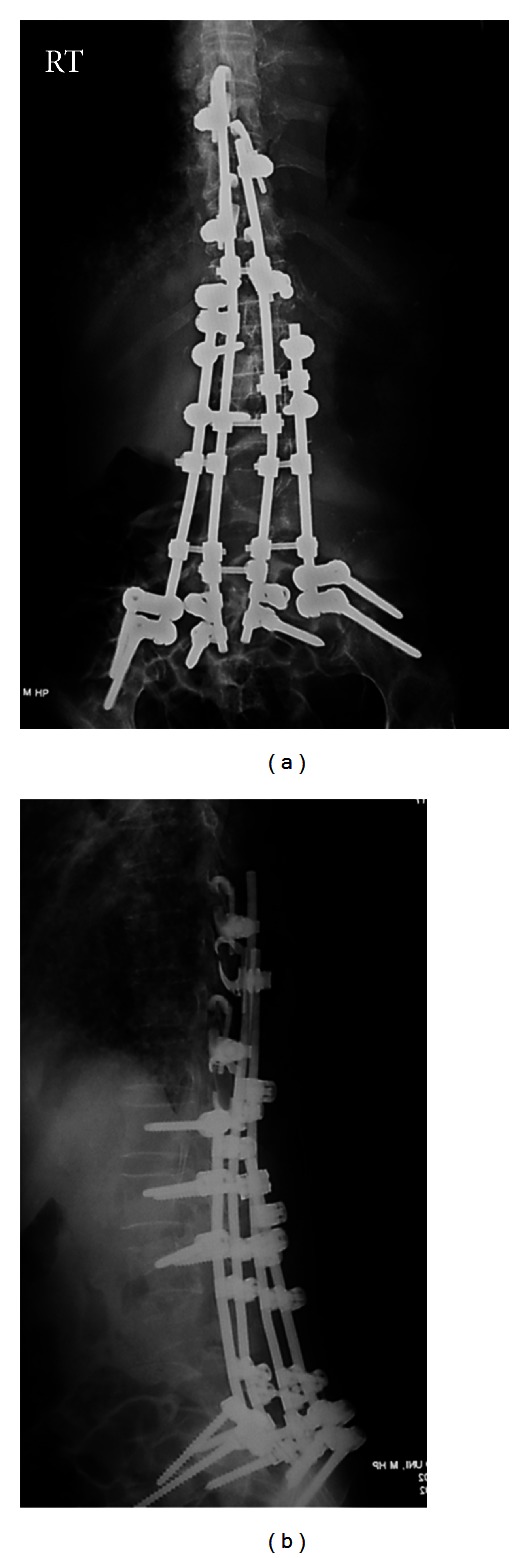
Plain radiographs after the second surgery (June 2010).

Sagittally reconstructed CT revealed the tumor had caused complete destruction of the L3, L4, and L5 vertebrae including the vertebral bodies and laminae (Figure 4). The mass was large and compressing the neural tissue at the L3, L4, and L5 levels (Figure 5). The pedicle screws inserted at L3 and L5 had completely loosened and were displaced (Figure 6). Motor weakness and sensory disturbance were noted in the areas of L3, L4, L5, and S1; however, the patient had normal bladder and bowel function. This time, metastasis was seen only in the spine and not in main organs such as brain, lung or liver. The patient was totally bedridden due to severe pain and paresis; therefore, we decided to restabilize the severely destabilized lumbar spine, and surgery was planned for June 2010. Embolization of the tumor feeding artery was conducted before the surgery. During the surgery, to avoid excessive bleeding, stabilization was completed first. For the upper segment of the implants, a laminar-laminar claw hook was selected. On the right side, the hooks were connected to the Th8 and Th10 laminae and on the left side to the Th9 and Th11 laminae. Pedicle screws were then inserted bilaterally at Th12, L1, and L2. For the lower segment of the implants, there were no normal vertebrae to use in the lumbar spine; thus, we decided on sacral and iliac screwing. S1 pedicle screws were bilaterally inserted in the inside direction, and S2 pedicle screws were inserted in the outside direction bilaterally. Two iliac screws were inserted in both iliac crests. There was a large bony gap between L2 and the sacrum, so we used 4 rods to stabilize the spine (Figure 7). For the outer rod, 2 iliac screws in the lower segment and 2 pedicle screws in the upper segment were connected bilaterally. For the inner rod, 2 sacral pedicle screws and 4 laminar thoracic hooks were connected. Then, each rod was connected by transverse cross-link devices. After these stabilization procedures, the large mass was partially resected by cavitational ultrasonic surgical aspiration until suture of the bilateral paravertebral muscles and skin closure were possible. Operation time was 7 hours 50 minutes, and intraoperative blood loss was 4200 mL. No surgery related complications were encountered. 

**Figure 8:**
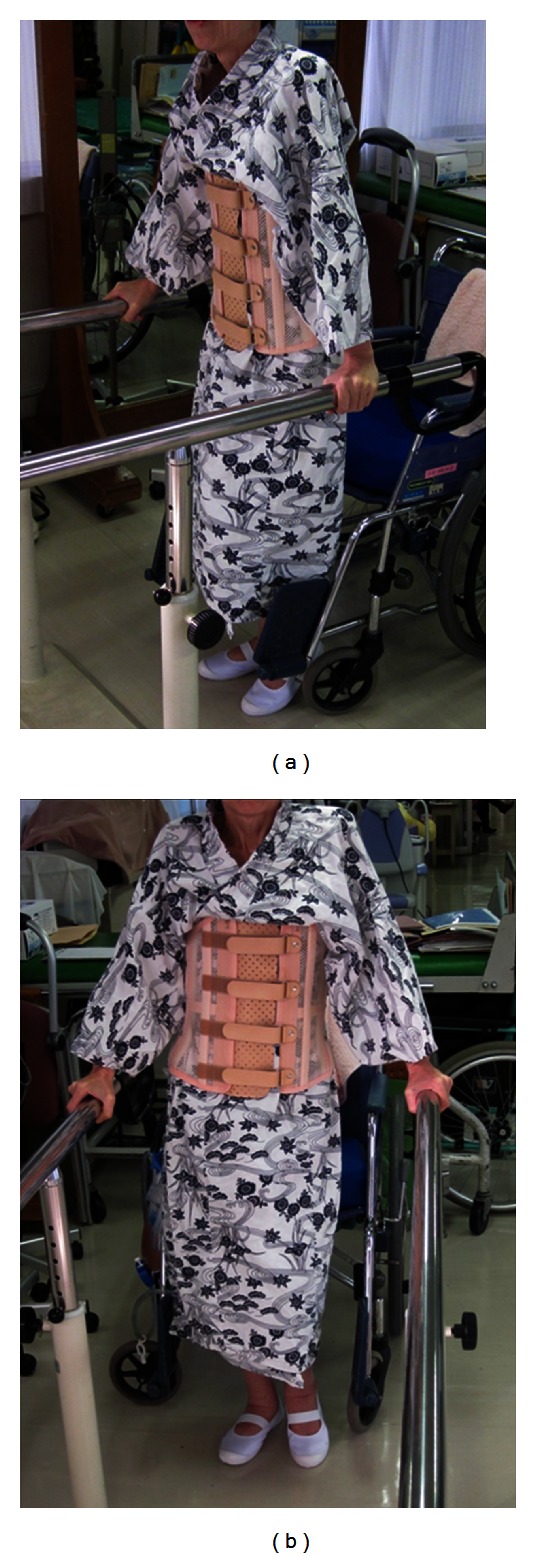
Patient at 3 months after the second surgery doing exercises for walking and standing (September 2010).

After the surgery, weakness was 0/5 on the manual muscle test (MMT) for the tibialis anterior, and extensor hallucis longus was not recovered bilaterally. The iliopsoas, quadriceps femoris, triceps surae, and flexor hallucis longus were maintained at MMT 4/5. Pain was gradually improved and 3 months after the second surgery, she started standing and walking exercises (Figure 8). Six months after the surgery, the patient died of lung metastasis. 

## 3. Discussion

It is well accepted and has been demonstrated by several authors that the primary tumor site is the most important prognostic factor for survival [1–3]. Since the primary lesion in the present case was a thyroid adenocarcinoma, this tumor is classed as a slow growing type according to the classification used by Tomita et al. [3]. According to Tokuhashi et al.'s scoring system [2], thyroid cancer has the highest and thus best score for good prognosis. Immediately before the first surgery in the current case, the Tokuhashi score was 12 and Tomita score was 5. Both scores indicate that the prognosis was more than 1 year, and therefore, we decided to stabilize the patient's unstable spine. Indeed, after the first surgery, the patient spent 2 years without intolerable pain or disability.

Just before the second surgery, Tomita's score was again 5, the same as before the initial surgery, but Tokuhashi's score had decreased to 9. The score at that time indicated that the patient had a prognosis of more than 6 months. Therefore, we decided to perform restabilizing surgery. Before the surgery, the patient was bedridden due to severe back and leg pain. After the surgery, as shown in Figure 8, she actively performed rehabilitation exercises for standing and walking. Her pain was controlled by medication such as narcotics. About 6 months after the second surgery, the patient died of lung metastasis.

The most challenging surgical problem in the present case concerned the complete destruction of 3 vertebral levels, which resulted in extreme destabilization. While there is no evidence to suggest that 2 rods would not be sufficient for stabilizing 3 vertebral levels, considered that 2-rod instrumentation could lead to rod stress fracture as a complication, Hedequist et al. [4] reported the usefulness of a 3-rod technique for correction of pediatric deformities, stating that the addition of the third rod increased the stability of the entire system and prevented instrumentation failure. In light of this, we decided to use 4 rods to stabilize the spine in our case (Figure 7). For each rod, we used at least 2 anchors including pedicle screws or laminar hooks for each end. The surgery obtained very strong stability, and the patient's quality of life was dramatically improved; whereas she had been previously bedridden, she was able to stand with support. Six months after the second surgery, the patient died and no instrumentation failure was observed. To the authors' knowledge, this is the first report to detail the use of a 4-rod technique for a spine with severe destruction. To understand the biomechanical effects of the technique, finite element analysis might be the best option, and there is a finite element model including the spine and pelvis available [5]. 

Surgical-related complications have been reported regarding metastatic spinal tumor [6–9]. Lau et al. [9] reviewe 106 cases who underwent spinal reconstruction surgery for metastatic tumor. They found overall complication rate was 21.7%. They also stated that patients older than 40 years old or patients who have metastatic lesions involving three or more contiguous vertebral levels would be higher risk for complication. In this case, patient was 67 years old and had 3 levels at L3-L4-L5, indicating higher risk having surgery-related complications. Thanks to team management during and after the surgery, we did not encounter any complications such as dural tear, nerve involvement, infection, pulmonary embolism, and so on. To minimize such complication, minimally invasive technique has been proposed [10, 11]; however, for such severely destructed cases like the current case, minimally invasive technique would not be realistic.

## Figures and Tables

**Table 1:** Neurological findings before the first surgery (January 2007).

	Right	Left
Sensory hypesthesia		
L4	+	−
L5	+	−
S1	−	−
MMT		
Quad	4	5
TA	5	5
EHL	5	5
FHL	5	5
Reflex		
PTR	++	++
ATR	++	++

**Table 2:** Neurological findings before the second surgery (June 2010).

	Right	Left
Sensory hypesthesia		
L4	+	+
L5	+	+
S1	+	+
MMT		
Quad	3	3
TA	0	0
EHL	0	0
FHL	3	3
Reflex		
PTR	++	++
ATR	++	++
